# The First Episode Psychosis Services Fidelity Scale as a Measure of Quality of Care

**DOI:** 10.1093/schizbullopen/sgaf030

**Published:** 2025-11-25

**Authors:** Su Lynn Tan, Julia Kirkham, Donald Addington

**Affiliations:** Department of Psychiatry, Cumming School of Medicine, University of Calgary, Calgary, AB T2R 0X7, Canada; Department of Psychiatry, Cumming School of Medicine, University of Calgary, Calgary, AB T2R 0X7, Canada; Hotchkiss Brain Institute, University of Calgary, Calgary, AB T2N 4N1, Canada; Department of Psychiatry, Cumming School of Medicine, University of Calgary, Calgary, AB T2R 0X7, Canada; Hotchkiss Brain Institute, University of Calgary, Calgary, AB T2N 4N1, Canada

**Keywords:** schizophrenia, psychoses, quality of care, scales/outcomes and performance measurement

## Abstract

**Background:**

Fidelity scales are designed to assess the degree to which a program delivers the core components of evidence-based programs. The degree to which scales can predict outcomes—its predictive validity—is an important psychometric property. Fidelity scales have multiple applications, including in quality assurance and improvement. The purpose of this study was to assess the degree to which the First Episode Psychosis Services Fidelity Scale (FEPS-FS) assesses the 6 domains of quality of care (QoC) described in the Institute of Medicine (IOM)’s QoC framework.

**Study Design:**

A quality thematic analysis using a theoretical (deductive) approach was used to categorize the 36 items of the FEPS-FS according to the framework for evaluating QoC outlined by the IOM. Two coders independently reviewed and coded each item in duplicate. Frequency counts were used to summarize the characteristics of the FEPS-FS items across the IOM’s quality domains.

**Study Results:**

Most FEPS-FS items reflect effectiveness (47.2%), efficiency (19.59%), and patient-centeredness (18.56%). Safety (7.22%), timeliness (5.15%), and equity (2.06%) were relatively underrepresented.

**Conclusions:**

The FEPS-FS can be considered as a measure of QoC that reflects all the IOM domains. As expected for a fidelity scale, the largest number of items assess effectiveness, while safety, timeliness, and equity were represented by fewer items. We identified potential items from the literature that could be used to increase the proportion of items in underrepresented quality domains in future iterations of the FEPS-FS or other fidelity scales.

## Introduction

Fidelity scales measure whether a mental health program or intervention is implemented as intended or adheres to an evidence-based care treatment model.^1^^,^^2^ Fidelity scales are developed by identifying the core components of an evidence-based program or intervention that assess structure and process and link processes or interventions to outcomes.^3^ In addition, they provide a list of objective criteria by which a program or intervention is judged to adhere to a reference standard, and they allow for benchmarking and comparisons across programs and services.^4^ The degree to which the level of fidelity is related to outcomes—its predictive validity—is an important psychometric quality of fidelity scales.^5^^,^^6^ However, fidelity scales must balance effectiveness with other important aspects of treatment due to the emergence of new frameworks for implementation research and quality improvement.^7^

The Institute of Medicine (IOM) framework for quality of care (QoC) provides a common language for understanding QoC based on empirical evidence, theory, and expert consensus.^8^ It identifies 6 key domains of healthcare performance that together constitute high QoC^8^ (Table 1). These QoC domains are used by leading national health agencies, such as the United States Agency for Healthcare Research and Quality and have been applied to mental health services,^8^^,^^9^ including for schizophrenia and early intervention services.^10^^,^^11^ Receipt of high QoC is particularly important for individuals with first-episode psychosis (FEP), as early intervention can mitigate negative health outcomes such as reducing the duration of untreated psychosis, preventing relapses, and slowing disease progression.^12^

**Table 1:** The Number of First-Episode Psychosis Service Fidelity Scale Items Representing Institute of Medicine Quality Domains

**IOM** ^**a**^ **quality domains**	**Definition**	**Summary and example of FEPS-FS** ^**b**^ **items**
Equity (*n* = 2)	Providing equitable and consistent care to all patient groups, regardless of personal characteristics such as gender, ethnicity, location, or socioeconomic background.	Program includes serving specific geographical populations and age groups. For example, program serves ages 14-65, the age of schizophrenia onset (Component #10: Age Range Served).
Safety (*n* = 7)	Delivering intended care that prioritizes patient safety while minimizing risk of harm.	Program includes antipsychotic dose monitoring, comprehensive risk assessments, metabolic screening, physical assessments, and smoking documentation. For example, program measures, records, and monitors weight, triglycerides, glucose, and hemoglobin A1C (Component #25: Supporting Health).
Effectiveness (*n* = 46)	Optimizing evidence-based treatment and services for those who need them while avoiding unnecessary interventions for those who will not benefit.	Program includes case management, CBT, psychoeducation, psychotherapy, proper antipsychotic prescribing (including clozapine use), and fidelity monitoring. For example, program includes professionals that deliver health services, psychotherapy, substance use management, family education, and support, psychoeducation, and pharmacotherapy (Component #3: Services Delivered by Team).
Patient-Centeredness (*n* = 18)	Ensuring that care is aligned with patient preferences, values, and needs.	Program includes family involvement, social services, psychosocial needs assessment, patient-engaged care planning, and access to supported education, employment and housing services. For example, program includes an initial psychosocial needs assessment on housing, employment, education, social support, financial support, family support, past trauma, and legal support (Component #17: Comprehensive Psychosocial Needs Assessment).
Timeliness (*n* = 5)	Reducing wait times and minimizing delays for both patients and healthcare providers.	Program includes early intervention, timely contact, post-discharge follow-up, and continuous care. For example, patients have face-to-face contact with a first-episode psychosis service provider within 2 weeks of discharge from the hospital (Component #33: Timely Contact after Discharge Hospital).
Efficiency (*n* = 19)	Eliminating waste in all forms, including resources, supplies, ideas, and energy.	Program includes care transition, case management, proper provider caseloads, and resource allocation. For example, program reaches a 20:1 target ratio of active patient-to-provider (Component #2: Patient-to-Provider Ratio).

a Institute of Medicine.

b First-Episode Psychosis Services Fidelity Scale.

Balanced improvement across all domains allows mental health services to better meet patient needs and deliver safer, more reliable, responsive, and coordinated care, with access to a full range of services that support their needs.^8^ Conversely, a narrow focus on improvement (or evaluation for the purpose of guiding improvement) can lead to a skewed understanding of QoC and programs, neglecting essential aspects of high QoC or driving unnecessary care and inefficiency. For example, an intervention may be designed to be safe and patient-centered, but if it lacks efficiency, it could lead to resource under or overutilization or implementation challenges. It is thus important that scales used to monitor and evaluate programs reflect a range of quality domains.

The First Episode Psychosis Services Fidelity Scale (FEPS-FS) is a fidelity scale that evaluates programs providing integrated, team-based care for FEP,^13^ known as Coordinated Specialty Care (CSC) in the United States. The FEPS-FS was developed using an established methodology^14^; first, possible indicators or critical components of a model based on research on effective programs were identified, then, using an iterative process, objective, and measurable definitions for these components were developed by clinician experts. Lastly, the psychometric qualities of the scale, such interrater reliability, predictive validity, and discriminative ability were evaluated. The FEPS-FS development process has been published elsewhere.^15^ The FEPS assesses all components of CSC with high interrater reliability and predicts outcomes.^15^^,^^16^ The FEPS-FS has been compared to the National Clinical Audit for Psychosis (NCAP), a set of quality measures that has been used annually at a national level to assess early psychosis intervention (EPI) services quality in the United Kingdom.^17^ The FEPS-FS items encompass all the indicators used in the NCAP and reflects the same evidence base. The FEPS-FS is flexible and adaptable for a number of purposes. It has been used as a binary measure in California to identify the presence or absence of core components of care across a large system,^18^ as a self-reported measure to evaluate national services in Italy,^19^ and to evaluate pilot programs in Czechia.^20^ In Canada, it has been used to assess QoC, rapidly evaluate service changes during COVID-19, and to test implementation strategies in FEP programs.^21–23^ Nationally in the United States, it has been used in a Learning Health Care Network and adapted to evaluate services for individuals at clinical high risk for psychosis.^24^^,^^25^

The objective of this study was to assess the FEPS-FS as a measure of QoC by determining the extent to which its items assess the quality domains identified by the IOM for the assessment of QoC.

## Methods

A quality thematic analysis was used to categorize the 36 items of the FEPS-FS according to the IOM quality domains.^26^ A preliminary coding structure was developed using a theoretical (deductive) approach.^26^

Individual items from the FEPS-FS (version 1.1) were exported to Microsoft Excel v16.88. Each item of the FEPS was then reviewed and coded in duplicate (SLT and JK) based on its definition, rationale, and item response rating according to the 6 core dimensions of healthcare quality outlined by the IOM.^8^ Coders were experienced in qualitative content analysis and knowledge synthesis research methods (SLT) and healthcare quality improvement measurement and methodology, as well as psychiatric clinical care (JK), informing the coding process, providing additional insight into the alignment of items with established quality domains.

Items were coded based on predefined criteria, based on definitions of quality domains, to ensure consistency between both coders (Table 1). For example, equity was assessed based on whether programs served specific geographical populations and age groups. Safety was evaluated based on the inclusion of scale items aimed at minimizing harm, such as antipsychotic dose monitoring, comprehensive risk assessments, metabolic screening, physical assessments, and smoking documentation. Effectiveness was coded when scale items were based on maximized delivering evidence-based treatment, incorporating aspects of case management, cognitive behavioral therapy, psychoeducation, psychotherapy, and appropriate antipsychotic prescribing. Patient-centeredness was determined by the extent to which scale items incorporated elements that prioritized patient preferences, values, and needs, such as family involvement, social services, psychosocial needs assessment, patient-engaged care planning, and access to supported education, employment, and housing services. Timeliness was coded based on scale items aimed at reducing wait times and delays, including early intervention, timely contact, post-discharge follow-up, and continuous care. Efficiency was assessed by whether scale items minimized waste and optimized resources. This included incorporating care transition, case management, appropriate caseloads, and resource allocation.

Some of the 36 FEPS-FS items are calculated by summing the number of features required of the item. For these FEPS-FS items, ratings for each distinct sub-item within the broader FEPS-FS item were coded according to the corresponding quality domain. For example, *Item #3: Services Delivered by Team* consists of eight distinct sub-items, each categorized as an individual quality domain, resulting in eight coded ratings. Overall, nine items (i.e., items #3, 6, 7, 16, 17, 25, 27-29) contained distinct sub-items, leading to a total of 97 coded items (Table 1). After blind coding, coders met to discuss and resolve discrepancies, revising codes based on consensus. When consensus could not be reached, a third reviewer—an expert in assessing the quality of early psychosis care—was consulted to clarify the scale item and provide interpretive guidance, after which the two primary coders made the final coding decision. Frequency counts were then used to describe the characteristics of the FEPS-FS by summarizing the distribution of individual items across the IOM quality domains.

## Results

**Figure 1:**
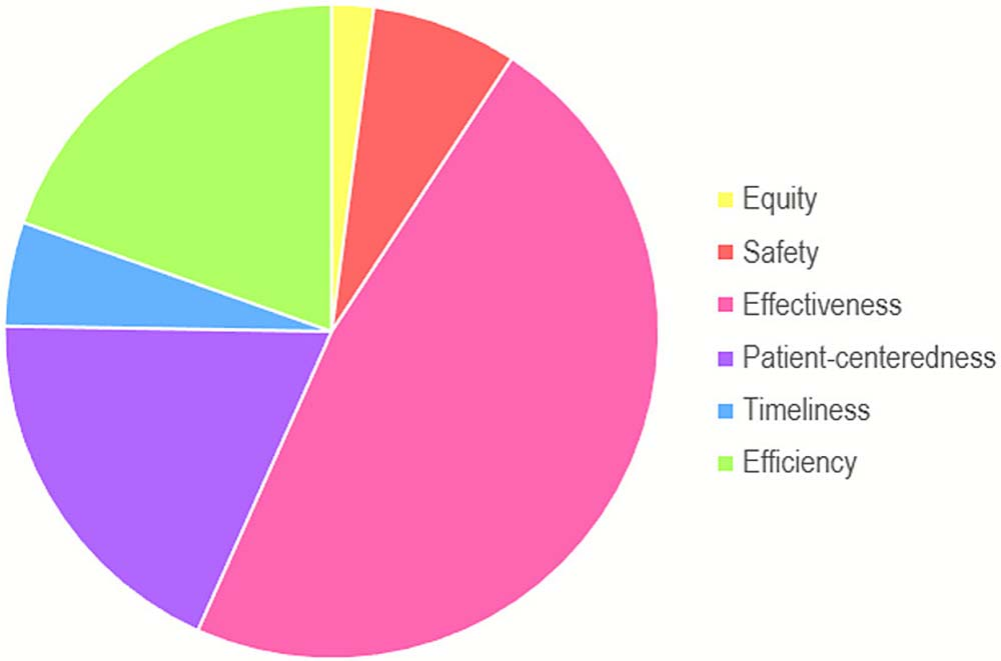
The Distribution of the 6 Domains of Healthcare Quality Indicators (Frequency Counts) Identified Through Thematic Analysis.

Among 36 items of the FEPS-FS, 97 total items (including sub-items) were coded (Table 1). Most FEPS-FS items were related to the effectiveness quality domain (47.42%; *n* = 46; scale items #1-9, 11-12, 16-17, 19-28, 32, 34), followed by efficiency (19.59%; *n* = 19; scale items #2-7, 13, 27-33, 36) and patient-centeredness (18.56%; *n* = 18; scale items #3, 15, 17-19, 22-23, 29, 35). Fewer items were related to safety (7.22%; *n* = 7; scale items #16, 20, 25), timeliness (5.15%; *n* = 5; scale items #12-14, 33, 36), and equity (2.06%; *n* = 2; scale items #9-10) (Figure 1; Table 2).

**Table 2:** The First-Episode Psychosis Services Fidelity Scale Items and Their Corresponding Descriptions

**Scale item**	**Title**	**Description**
1	Practicing Team Lead	Team leader has administrative and supervisory responsibilities and provides direct clinical services.
2	Patient-to-Provider Ratio	Target ratio of active patient- to-provider (ie, team members) is 20:1.
3	Services Delivered by Team	Qualified professionals deliver services that include the following: (1) Case management/care coordination; (2) Health services; (3) Psychotherapy; (4) Substance use management; (5) Supported employment; (6) Family education/support; (7) Patient psychoeducation; (8) Pharmacotherapy.
4	Assigned Case Manager/Care Coordinator	Patients have an assigned clinician who delivers case management services/care coordination.
5	Psychiatrist/Prescriber Caseload	Each patient has an assigned psychiatrist/prescriber who has a caseload that allows them to see patients for medication reviews or other clinical indications.
6	Psychiatrist/Prescriber Role on Team	Psychiatrists/prescribers are team members who: (1) attend team meetings; (2) see patients with other clinicians; (3) are accessible for consultation by the team during the work week; (4) shares health record with other team members.
7	Weekly Team Meetings	All team members attend weekly meetings that focus on (1) case review (admissions and caseloads); (2) assessment and treatment planning; (3) discussion of complex cases; (4) discharge (transition) planning.
8	Explicit Diagnostic Admission Criteria	Program has clearly identified mandate to serve specific diagnostic groups with a psychosis and uses measurable and operationally defined criteria to select patients. This includes a consistent process for including and documenting uncertain cases and those with co-morbid substance use.
9	Population Served	Program has a clearly identified mandate to serve a specific geographic population and uses a comparison of annual incidence to number of accepted cases of people with schizophrenia spectrum disorder to assess success in admitting all incident cases.
10	Age Range Served	Program serves the age spectrum from ages 14-65.
11	Duration of FEP Program	Formal funding mandate and policy of FEP program is to provide service to all patients for a specified period measured in years.
12	Targeted Education to Health/ Education/Social Service/Community Groups	The program operates a targeted active outreach program of educational workshops to groups of first-contact individuals in health, education, and social agencies, as well as community organizations.
13	Early Intervention	Team measures early intervention by the proportion of people hospitalized prior to FEPS admission.
14	Timely Contact with Referred Individual	Individuals with a first episode of psychosis commence treatment in early/first-episode psychosis services, as measured by time from referral to first face to face/video appointment with a clinician.
15	Family / Support Persons Involvement in Assessments	Service engages family/support person in initial assessment to improve quality of assessment and engagement.
16	Comprehensive Clinical Assessment	Initial clinical assessment includes: (1) time course of symptoms, change in functioning, and substance use; (2) recent changes in behavior; (3) assessment of risk to self/others; (4) mental status exam; (5) psychiatric history; (6) premorbid functioning; (7) co-morbid medical illness; (8) co-morbid substance use; (9) family history.
17	Comprehensive Psychosocial Needs Assessment	Initial psychosocial needs assessment includes: (1) housing; (2) employment; (3) education; (4) social support; (5) financial support; (6) primary care access; (7) family support; (8) past trauma; (9) legal.
18	Clinical Treatment/Care Plan after Initial Assessment	Patients, family/support person, and staff collaborate to develop a treatment/care plan that addresses clinical and psychosocial needs. The program engages patients in care planning by asking them to sign-off on plan.
19	Antipsychotic Medication Prescription	Psychiatrist/Prescriber prescribes antipsychotic with consideration given to patient’s preferred medications after assessment confirms a diagnosis of a psychosis.
20	Antipsychotic Dosing within Recommendations for Individuals with Psychosis	Six months after commencing treatment in FEPS, antipsychotic dosing is within government-approved guidelines for second-generation antipsychotic medications, and between 300 and 600 chlorpromazine equivalents for first-generation antipsychotics.
21	Clozapine for Medication-Resistant Symptoms	Use of clozapine if an individual with schizophrenia spectrum disorder (SSD) does not respond to 2 courses of first-line antipsychotic medication.
22	Patient Psychoeducation	Patients receive at least 8 sessions of patient psychoeducation/illness management training in the first year. Delivered by trained clinicians, either to individuals or in group psychoeducation sessions.
23	Family/Support Person Education and Support	Family/Support Person receives at least 8 sessions of evidence-based individual or group family education and support that covers curriculum in the first year of patient being in the program. Education and support provided by a clinician trained to deliver the program.
24	Cognitive Behavioural Therapy (CBT)	Patients receive at least 10 sessions of CBT delivered in individual or group format in the first year of the program. Delivered by an appropriately trained clinician, for indications such as positive symptoms, anxiety, depression, or trauma.
25	Supporting Health	Program takes steps to support patient health through the following: (1) refer and enroll patient in primary care; (2) Measure and record weight at least quarterly in first year of program; (3) provide feedback on weight gain and advice on diet and exercise; (4) monitor and document extrapyramidal side-effects; (5) Monitor triglycerides and glucose/Hb A1c annually; (6) monitor and document cigarette smoking habits; (7) prescribe pharmacological support to smokers/vapers wishing to quit.
26	Annual Assessment	Includes documented assessment of: (1) educational involvement; (2) occupational functioning; (3) social functioning; (4) symptoms; (5) psychosocial needs; (6) risk assessment of harm to self or others; (7) substance use.
27	Services for Patients with Substance Use Disorders	FEP program offers the following: (1) routine assessment of substance use for all patients at intake and at review; (2) substance use addressed in patient psychoeducation; (3) substance use addressed in family psychoeducation; (4) brief evidence-based psychotherapies including motivational enhancement or CBT for patients with substance use problems; (5) continuity of care and patient engagement for patients referred to specialized substance use services ranging from detox to residential treatment.
28	Supported Employment (SE)	Program provides SE to patients interested in participating in competitive employment. Elements of SE include: (1) trained SE specialist with at least 6 months experience; (2) SE specialist is a FEPS team member and attends team meetings; (3) SE specialists receive at least twice monthly supervision from a qualified supervisor; (4) ratio of SE specialist caseload is 1:20 or less; (5) SE has ≥6 employer contacts per week; (6) uses career profile or equivalent; (7) tracks in-person employer contacts.
29	Supported Education (SEd)	Program provides SEd to patients interested in participating in education as evidenced by (1) a designated SEd specialist; (2) SEd specialist is part of FEPS team; (3) SEd caseload of at least 3 patients with education goals; (4) SEd specialist completes and documents educational goals. Support Items: SEd specialist supports patients to: (1) explore education programs; (2) secure sources of financial aid; (3) complete applications and enrollment; (4) manage course work; (5) identify legislated and other sources of support for high school students.
30	Active Engagement in the Community	Designated program staff use outreach including community visits to engage individuals with FEP.
31	Patient Retention	Program measures patient retention using the ratio of the number of patients who left the program during their first year for any reason in the last 12 months to the total current caseload.
32	Crisis Intervention Services	FEP service providers either deliver crisis services or have formal links to crisis response services that include crisis lines, mobile response teams, urgent care centers, or hospital emergency rooms
33	Timely Contact after Discharge from Hospital	Patient in FEP service have face-to-face or video contact with FEP service provider within 2 weeks of discharge from hospital.
34	Assuring Fidelity	Program monitors quality using a published fidelity scale or quality indicators linked to standards for program treatment components calculated from health record audit or administrative data.
35	Peer Support Specialist: Role on Team	Peer Support specialist may improve patient experience and quality of life. They can fulfil a number of roles from outreach and retention to leading recovery groups. Peer Support workers should: (1) have direct lived or living experience of psychosis; (2) have training to deliver the service they provide to patients; (3) have a program of support and supervision within team; (4) have peer support training from a recognized provider of peer support training; (5) have funding from team or agency budget; (6) attend team meetings.
36	Transition in Care	Team plans for transition to an appropriate level of care 6-12 months prior to time of discharge. Program sends a referral to follow-up services prior to discharge, documents both the service to provide follow-up and a scheduled appointment.

## Discussion

This study demonstrated that it is possible to examine a fidelity scale from the perspective of a broad definition of QoC. This qualitative content analysis also demonstrates that the FEPS-FS reflects all the IOM domains of QoC. As expected, the IOM domain of effectiveness represented the highest proportion of items. Equity, however, was the least represented. Safety and timeliness were covered by a slightly higher proportion of items.

Effectiveness is often prioritized in quality measurement and mental health services monitoring. Regulatory and funding agencies often favor effectiveness as a primary healthcare objective due to its relative ease of measurement using metrics such as hospital readmission and mortality and its historical alignment with traditional medical goals.^27^

The high representation of patient-centeredness in the FEPS-FS is noteworthy, as this quality domain has historically received less attention in mental health services performance or quality measurement; however, it is increasingly recognized as an important aspect of high QoC.^28^^,^^29^ Although many fidelity scales assess whether interventions align with patient values or individualized treatment goals, recently fidelity scales have incorporated more comprehensive patient-centered elements, such as shared decision-making between patient and providers to guide various aspects of care.^30–32^

Equity is a domain that has been poorly defined within healthcare until recently and often overlooked as a key indicator of good QoC.^33^ Inequities in care (eg, variable access to care based on age, race, gender, location etc.) compared to the general population are common in schizophrenia and FEP and an important contributor to poor outcomes.^34–36^ For example, women and people of color are most vulnerable to medical undertreatment in schizophrenia.^37^ Racial and ethnic minority groups often encounter additional barriers and longer delays before receiving care compared to their White counterparts.^35^^,^^38^ Similarly, individuals from linguistic minority groups may struggle to engage with services that lack interpretation or culturally adapted services.^39^

Many fidelity scales do not evaluate whether programs are delivered across geographical, racial, or socioeconomic groups, which risks enforcing disparities in current programs or interventions.^40^^,^^41^ The Health Equity Measurement Framework provides a framework to evaluate whether programs are delivered equitably.^42^ Embedding equity considerations into fidelity scales can ensure that programs and interventions reach and engage diverse populations. Items that identify whether the population served within a program reflects the distribution of equity considerations in the larger population and/or can be developed.^42–45^

The FEPS-FS incorporates equity items related to the population serviced (scale item #9) and the age range served (scale item #10). These components are important because EPI programs often disproportionately engage younger males.^46^ Despite this strength, the FEPS-FS, like other fidelity scales, does not address several important and emerging dimensions of equity, including accessibility of services for a diversity of racial, ethnic, and language populations with FEP. Incorporating additional specific fidelity items in future iterations of the FEPS-FS, such as items assessing cultural or ethnic identity of individuals enrolled in FEP programs and availability of tailored resources, could assess the degree to which programs admit and serve patients that reflect the diversity of the population that they serve.

Efficiency is also a critical item of fidelity scales, as rigid adherence to evidence-based models may be impractical in real-world settings due to workforce constraints, funding limitations, and other systemic challenges faced by healthcare programs.^47–49^ Evaluating whether mental health service program fidelity can be maintained without unnecessary resource strain or waste is essential to ensuring that high QoC is sustainable and effective.

We found no past studies that assessed the comprehensiveness of fidelity scales from the perspective of QoC domains. Many fidelity and performance measurements scales focus on specific domains and may not reflect a range of important care processes and services in schizophrenia or FEP. For example, the Physical Health Care Fidelity Scale has a narrower focus, targeting physical health policies that support health outcomes for individuals with psychosis, and is based primarily on safety, defined as avoiding harm to patients from the medical care intended to help them.^8^^,^^50^ Items of the Physical Health Care Fidelity Scale have not been thoroughly assessed within the full context of healthcare quality.^50^ In fidelity scales that do explore these domains, the focus is often on effectiveness and efficiency, which have historically been prioritized as key measures.^7^^,^^51^ Given these challenges, fidelity scales for programs serving individuals with schizophrenia or FEP can be refined to better assess equity, patient-centeredness, and efficiency, ensuring that interventions are not only implemented as designed but also applicable across diverse settings.

In addition to the content of fidelity scales, provider perspectives on use of them is important to consider. Although many clinicians express a strong interest in quality improvement, some regard program fidelity monitoring as overly rigid or restrictive of professional autonomy.^52^^,^^53^ However, evidence indicates that when program fidelity monitoring is implemented effectively, it can motivate providers and enhance quality improvement.^52^ A Canadian study in which senior clinical providers of EPI services were trained to assess the fidelity of programs other than their own describe the process in positive terms, such as validating their work and inspiring efforts at quality improvement.^54^ Therefore, fidelity scales should aim to balance clarity and specificity about what constitutes good QoC with the flexibility for providers to adapt to different situations and exercise their professional judgement.

A number of fidelity scales have adapted the FEPS-FS with the stated goal of reducing the cost and burden of fidelity assessment, adapting the scale to local circumstances and focusing on a specific model of service delivery.^24^^,^^55^^,^^56^ An overview of these adaptations identified that they had not published data to support cost and reductions compared with published data for the FEPS-FS.^15^

As evidence and understanding of best practices in healthcare evolve, evaluation methods should adapt accordingly, balancing comprehensive assessment with practical considerations, such as availability of data and feasibility of collection. High QoC is increasingly recognized as care that reflects a range of quality domains, in turn, to drive improvement and ensure effective evaluation, methods for evaluating QoC and service performance should ensure that these domains are reflected to provide a more thorough evaluation of healthcare quality.

### Strengths and limitations

This analysis offers valuable insights through a structured, theory-driven approach.^22^ A key strength is the use of deductive content analysis based on the IOM quality domains, which provides a clear coding framework and minimized bias through predetermined categories.

However, several limitations should be acknowledged. While the coders (SLT and JK) brought relevant expertise in healthcare quality, their familiarity with the subject may have introduced bias. To mitigate this, the process included blind coding, a structured consensus process and expert adjudication (DA). Some domains, such as patient-centeredness, are inherently more subjective.^33^ The use of clearly defined and widely recognized QoC domains from a leading agency, the IOM, supported consistency and reduced interpretive variability between coders. Broadly speaking, while the deductive approach was systematic, it may have constrained interpretation by requiring all data to align with predefined categories, potentially limiting opportunities for deeper exploration.

## Conclusion

This study has demonstrated that this approach to evaluating fidelity scales is feasible. We found that the FEPS-FS addresses all 6 healthcare quality domains identified by the IOM. However, as is typical of fidelity scales, the FEPS-FS items emphasize effectiveness of care services. A significant strength of the scale is the extent to which it addresses patient-centeredness. Domains that were covered by a lower proportion of items could be strengthened by incorporating additional evidence supported items and rating scales to improve comprehensive coverage of QoC in future iterations. Findings from this fidelity scale evaluation may help inform the development and refinement of similar measurement tools in the future.
